# A review on powder-based additive manufacturing for tissue engineering: selective laser sintering and inkjet 3D printing

**DOI:** 10.1088/1468-6996/16/3/033502

**Published:** 2015-05-05

**Authors:** Seyed Farid Seyed Shirazi, Samira Gharehkhani, Mehdi Mehrali, Hooman Yarmand, Hendrik Simon Cornelis Metselaar, Nahrizul Adib Kadri, Noor Azuan Abu Osman

**Affiliations:** 1 Department of Mechanical Engineering and Advanced Material Research Center, University of Malaya, 50603 Kuala Lumpur, Malaysia; 2 Department of Biomedical Engineering, Faculty of Engineering, University of Malaya, 50603 Kuala Lumpur, Malaysia

**Keywords:** additive manufacturing, inkjet 3D printing, selective laser sintering, biomaterials, tissue engineering

## Abstract

Since most starting materials for tissue engineering are in powder form, using powder-based additive manufacturing methods is attractive and practical. The principal point of employing additive manufacturing (AM) systems is to fabricate parts with arbitrary geometrical complexity with relatively minimal tooling cost and time. Selective laser sintering (SLS) and inkjet 3D printing (3DP) are two powerful and versatile AM techniques which are applicable to powder-based material systems. Hence, the latest state of knowledge available on the use of AM powder-based techniques in tissue engineering and their effect on mechanical and biological properties of fabricated tissues and scaffolds must be updated. Determining the effective setup of parameters, developing improved biocompatible/bioactive materials, and improving the mechanical/biological properties of laser sintered and 3D printed tissues are the three main concerns which have been investigated in this article.

## 1. Introduction

Additive manufacturing (AM) is a technique for fabricating parts in precise geometry using computer aided design (CAD) and computer aided manufacturing (CAM) [1]. In each AM technique, the 3D model designed in CAD software is converted to STL format, which is a triangular mesh of the object, and then the STL format is sliced into 2D profile layers. Each sliced layer of the model is bonded to the previous layer on the build platform until a 3D part is fabricated. The principal AM technologies are selective laser sintering (SLS), stereolithography (SLA), fused deposition modeling (FDM), direct metal laser sintering (DMLS), and inkjet 3D printing (3DP) techniques [2, 3]. Depending on the process and materials used, each technique has both strong and weak points. The most significant elements that should be considered in choosing an appropriate AM technology for a particular purpose are accuracy, time, and cost of fabrication. The parameter of accuracy refers to the thickness of the layers and the system of consolidation, and since AM techniques are tool-free fabrication methods, time of production can outweigh increased fabrication costs per item [3, 4].

Biomedical applications, e.g., tissues, scaffolds, and fixation devices, have specific aspects of fabrication which should be considered. For biomedical applications, the use of these AM methods without rigid support structures is strongly recommended [5]. In supportless AM methods the imprinted powders surround and support complex parts during the printing process, and after finishing the process, users can reuse all uncured support powders. Other additive processes require the building of solid support structures to support complex geometries during the printing process. Users have to discard these support structures after use, and the wasted material contributes significantly to the cost of additive technologies. In addition, removing attached supports from fabricated parts limits the ability to stack or nest parts [6].

AM approaches, particularly 3DP and SLS, are simple and adaptable to using a broad range of powders to produce porous ceramics, polymers, and metal-based tissues [7, 8]. To enhance bone regeneration in fabricated tissues, using powder-based AM techniques is recommended. These kinds of fabricated scaffolds can be filled with a porous spacer, allowing the ingrowth of a blood vessel [9].

In this article, the working principle of SLS and inkjet 3DP and modifications of these methods are reviewed. Materials used in SLS and inkjet 3DP and optimization of the effective parameters of these two powder-based AM techniques for the fabrication of useful bone tissues and scaffolds are highlighted. Biological tests (*in vitro*, *in vivo* and apatite layer formation) conducted on the fabricated tissues and scaffolds are presented and discussed, as well as clinical works regarding fabricated objects.

## 2. Laser sintering technology

**Figure 1.**
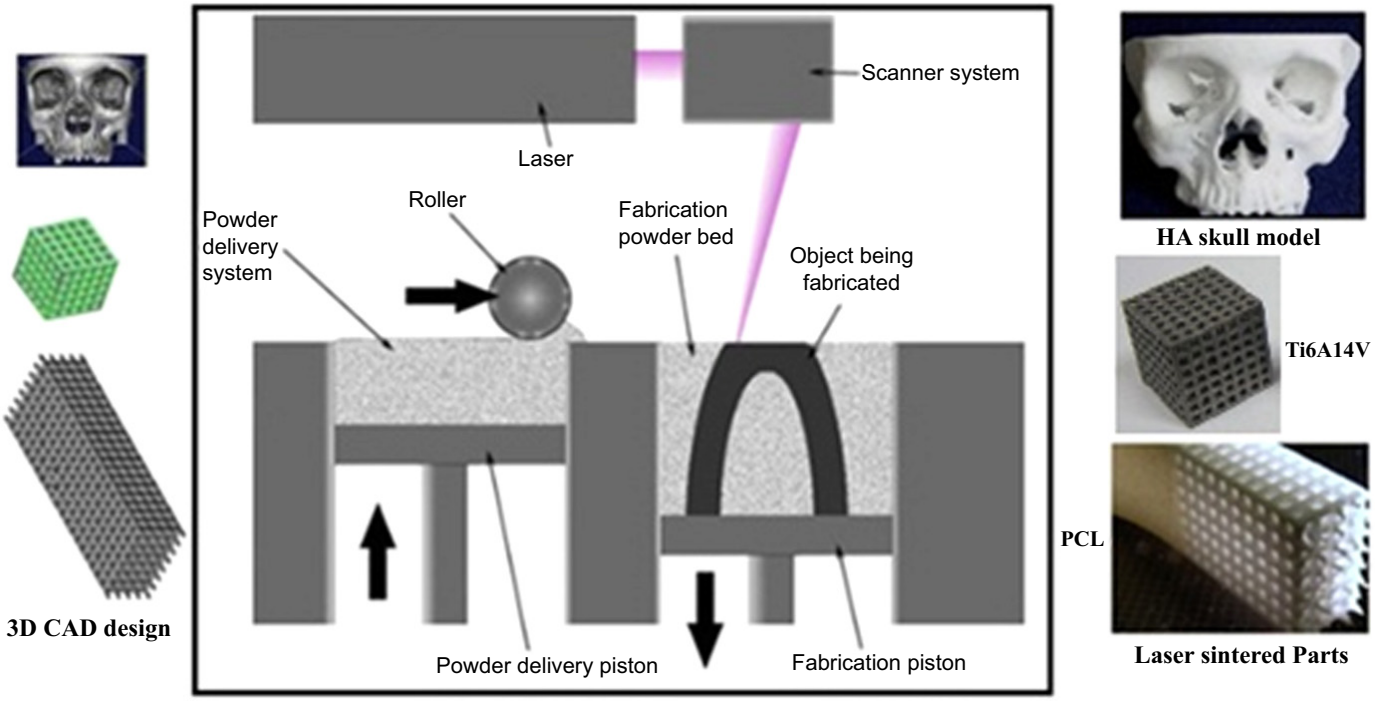
Schematic of SLS from 3D CAD design to the laser sintering process. Reprinted from D N Silva 2008 *J. Cranio-Maxillofacial Surg.* **36** 443–9, Copyright 2008, with permission from Elsevier; S Eshraghi and S Das 2010 *Acta Biomater.* **6** 2467–76, Copyright 2010, with permission from Elsevier; and E Sallica-Leva *et al* 2013 *J. Mech. Behav. Biomed. Mater.* **26** 98–108, Copyright 2013, with permission from Elsevier.

The SLS technique as depicted in figure 1 uses a CO_2_ or Nd:YAG laser beam for scanning successive layers of powdered materials to create a 3D object [10]. Based on slicing of the digital design, the scanning patterns of each layer are computed automatically [11]. As is illustrated in figure 1, fabrication of the final parts using the SLS method includes two steps: 3D CAD design of the concept and transfer of the CAD data to the SLS machine to carry out fabrication with the desired powders.

Each AM system has a unique binding mechanism to bind the layers. The binding mechanism of SLS technology can be classified into three main categories [13, 15].

• Solid-state sintering, which is a thermal process. The binding mechanism in this category occurs between *T*_m_/2 and *T*_m_, wherein lies the melting temperature of the material in question.
• Liquid phase assisted sintering, which is commonly used for materials that are difficult to sinter. Liquid phase assisted sintering is the process of adding an additive to the powder which will melt before the matrix phase. This method is widely employed for fabrication of 3D parts from ceramic materials with incorporation of a small amount of polymers which will gradually decompose and completely disappear [16].
• Full melting, which is used for metallic and ceramic materials more than polymers. In this mechanism, near full density is reached in one step by melting the powders completely by laser beam, thus avoiding lengthy post-processing steps.

In the SLS method, material properties and process factors such as laser energy density, part bed temperature, layer thickness, and hatch distance affect the structural and mechanical properties of fabricated parts [17, 18].

In this AM technique particle sizes in the range of 10–150 *μ*m are preferred [19]. The ideal laser energy density follows from the melting point of the binders (in the liquid phase sintering method) or powders (in the full melting technique) and can be set by adjusting the laser power and scan speed [20].

By decreasing the laser scanning speed, denser parts may be obtained. This is caused by the longer interaction time between the powder and the laser beam, which boosts the rate of energy delivered to the powder bed [21]. A higher laser scan speed results in less energy transferred to the materials [22], leading to less sintering and in turn to more porosity. It should be noted that this case occurs especially in low melting point systems. Increasing the energy delivered to the powder bed promotes better melting of the powders, enabling more liquid phase to flow and infiltrate into the voids between the particles, which can lead to a denser structure [21].

On the other hand, sufficiently high energy density leads to the complete melting of the binder, which reduces material delamination and increases the density of the fabricated parts. Although the higher energy density increases the mechanical properties of the final parts, it sometimes leads to inaccurate dimensions [17]. As a practical matter, because the time of exposure of the material to the laser beam is too short, fabricating high-density parts is difficult. It is reported that an isothermal process as a second step using a furnace with a lower temperature than that obtained under the laser beam improves the density of the final parts [23].

Tan *et al* [24] have also conducted some preliminary laser sintering tests to determine the range of suitable processing parameters used in the SLS system. In their study, only one layer of material with 0.1 mm thickness was sintered to determine the parameter setting. First, they set the bed temperature to 110 °C and reported the formation of necks between particles at a laser energy of 12 W; however, there was delamination between the specimen layers. To improve the quality of the specimens, a higher bed temperature was used. In this study the optimum SLS processing factors were found to be 140 °C for the bed temperature and 12 W for the laser energy. For the materials with lower density and lower melting point, the applied laser power was lower [19, 25, 26].

The effect of layer thickness on the open porosity of parts fabricated by SLS has been studied by Salvani *et al* [20]. The results demonstrated that layer thickness has the greatest impact on the average pore width and on the proportion of pores with a proper size to facilitate bone regeneration. This phenomenon can be caused by thicker powder layers allowing less fusion between particles, resulting in less densification and higher open porosity. Table 1 summarizes the effect of layer thickness on the average pore size of fabricated SLS samples.

**Table 1.** Effect of layer thickness on average pore width and proportion of pores of a suitable size in SLS [20].

	0.15 mm thickness of each layer			0.17 mm thickness of each layer			0.19 mm thickness of each layer		
Laser power (W)	Average pore width (*μ*m)	Range pore width (*μ*m)	Porosity (%)	Average pore width (*μ*m)	Range pore width (*μ*m)	Porosity (%)	Average pore width (*μ*m)	Range pore width (*μ*m)	Porosity (%)
3.2	61	10–318	19	75	10–382	28	80	10–500	29
5.5	66	10–462	21	78	10–409	30	83	10–364	32
7.7	64	10–464	22	77	10–382	31	80	10–473	33
10	67	10–391	21	77	10–482	31	80	10–482	32

Similar results for the influence of layer thickness on the porosity and layer bonding have been obtained in other studies [21]. It was concluded that smaller layer thickness leads to stronger bonding between the layers and decreases the porosity of the parts. Finding an optimum layer thickness is necessary depending on which application is desired. The minimum layer thickness that can be used effectively is determined by the maximum particle size of the powder. If a too-small layer thickness is chosen, the blade will drag non-melted large particles or chunks of melted particles, displacing the previous sintered layers from their position. Consequently, layer thickness for denser product must be set to the minimum layer thickness and vice versa [21].

Hatch distance is another important parameter with respect to the properties of the parts fabricated by SLS. It has been confirmed that with a large increase in hatch distance in the prototype, there are dramatic changes in pore channels in its structure [27]. The different microstructure resulting from a large hatch distance can be explained by the overlapping theory. Overlapping addresses to what degree a new laser line scans over the previously scanned track. Decreasing the hatch distance brings the scan lines closer to one another until they overlap. As an example, if the laser beam spot size is 0.4 mm, the parts processed with a hatch distance less than the laser spot size (e.g., 0.1, 0.2, and 0.3 mm) have different degrees of overlap. A large part of the laser spot may scan over a previously scanned line and accordingly increase the flowing and spreading of the liquid between adjacent scan lines, which leads to an enhancement of the inter-line bonding and a reduction in porosity. When a hatch distance of 0.4 mm is chosen, no overlapping is observed, resulting in appropriate connectivity of the matrix and a more porous part [28].

Another phenomenon which can affect the surface morphology of samples fabricated by SLS is balling [29, 30]. Balling is defined as an agglomeration of the particles, occurring where the liquid phase breaks up into a row of spheres to reduce surface energy. The main factor leading to balling is the Gibbs–Marangoni effect, which is the mass transfer along an interface between two fluids due to the surface tension gradient [31]. In terms of temperature association, this phenomenon is also called thermo-capillary convection. Balling has a direct effect on creating large pores but is not a definitive solution for fabricating tissues with desired pores. Early experiments in using the SLS method for the fabrication of metallic parts confirmed balling during the process [32]. To diminish the balling effect and consequently to have a uniformly sintered specimen, not only do the SLS parameters need to be set, but multiphase powders need to be designed by mixing different materials with various melting temperatures or by employing a pre-alloyed powder system in which melting takes place over a temperature range [33, 34].

### 2.1. Commonly used materials in SLS

#### 2.1.1. Polymers

Two types of thermoplastics are used in SLS: semi-crystalline and amorphous [35]. An amorphous material has chain molecules arranged in a random manner, and semi-crystalline material has chain molecules arranged in an orderly structure. Semi-crystalline and amorphous materials have different thermal properties which determine the fabrication parameters in SLS.

The most important characteristics that determine the application of thermoplastic polymers are the glass transition temperature, *T*_g_, and the melting temperature, *T*_m_. The glass transition temperature *T*_g_ is the temperature where a rapid decrease in *E* (elastic modulus) occurs. It can be observed in amorphous material. Melting does not occur until the polymer reaches a higher temperature, *T*_m_. Below *T*_g_, the polymer is in the glass state and the molecular motion along the chain is frozen. When the temperature rises from *T*_g_ to approximately (*T*_g_ + 30 K), the molecular motion increases, causing the modulus to drop. Just above *T*_g_, the polymer behaves like a highly viscous liquid in which the chains are all tangled up with their neighbors [36, 37].

It has been also reported that a majority of semi-crystalline polymers have a glass transition temperature (*T*_g_) below or close to room temperature (−100 to 50 °C) and a melting temperature (*T*_m_) above 100 °C (between 100 and 400 °C) at which a considerable volume change occurs. On the other hand, amorphous polymers do not have a characteristic melting temperature range [38]. They have a *T*_g_ of ∼100 °C, above which the material will progressively evolve to a leathery, rubbery, and finally liquid state as the temperature increases, with no obvious transitions [38–40]. It is important to mention that both *T*_g_ and *T*_m_ depend directly on molecular weight. This is why a different setup is needed to run an SLS system for different thermoplastic materials.

As mentioned, the power of the laser applied in an SLS system has an important effect on the mechanical properties of the fabricated models. For a semi-crystalline polymer powder, laser consolidation occurs by heating it to above its *T*_m_ since semi-crystalline powders have a molecular structure with spiky melt points. They do not gradually become softer with a temperature increase and remain hard until a given quantity of heat is absorbed and then quickly change into a viscous liquid. Shrinkage often happens simultaneously with freezing. To minimize this drawback, it is better to preheat the powders and to keep them in a furnace below their melting temperature for several hours [38].

Consolidation of amorphous polymer powder happens by laser heating over *T*_g_, at which point the polymer is in a much more viscous position than semi-crystalline polymers at a similar temperature [41]. Unlike semi-crystalline polymers, amorphous polymers do not have a spiky melt point and soften slowly as the temperature rises. The viscosity of these materials changes when heated, but they seldom are as easy flowing as semi-crystalline materials.

There are a number of studies on using natural and synthesized polymers in SLS. For example, cellulose, the most abundant natural polymer [42], has been used to fabricate SLS scaffolds [18]. An important synthetic biodegradable polymer material is polycaprolactone (PCL) This material is semi-crystalline with high thermal stability and a degradation period of approximately two years [43]. Due to the good biocompatibility, bioresorbability, and processability of PCL, this polymer is used for tissue engineering [25] and cartilage repair [44–46].

#### 2.1.2. Ceramics

SLS of ceramic materials can be either direct or indirect. Direct SLS of ceramics can be powder based or slurry based. In the powder-based method, the packing density of the powder layers is low, leading to a lower sintered density and also leading to cracks due to thermal stresses in the parts [47]. Efforts have been made to develop direct SLS to produce fully dense ceramic composites [48]. In this method high laser energy is applied to a preheated powder bed, causing the powder to melt and avoiding thermal stress cracking.

On the other hand, slurry-based direct SLS takes advantage of more homogeneous and much more densely packed powder layers obtained from the slurry process. The concern is that this method produces parts with lower strength due to thermal cracks and microstructural inhomogeneities [49, 50].

Agglomeration of powders is a concern with using slurry-based SLS. An effective way to avoid agglomeration during laser sintering may be to process at a lower scanning speed or to employ a surfactant in a very low concentration [51]. Using a surfactant helps obtain a homogeneous green part which can demonstrate better mechanical properties. This method is appropriate when the purpose is the fabrication of ceramic scaffolds with bioactive ceramics such as calcium silicate and hydroxyapatite. Calcium silicate (CS, CaSiO_3_) is a bioactive ceramic that has been explored for tissue engineering applications over the last two decades [52–55]. Many studies have shown that CS is able to form an apatite layer on its surface by soaking in simulated body fluid (SBF) [55]. CS scaffolds with an interconnected pore structure can be made by SLS [56]. Another commonly used ceramic in tissue engineering and the SLS method is hydroxyapatite (HA, Ca_10_(PO_4_)_6_(OH)_2_). HA is a calcium phosphate ceramic (CP) material that is biocompatible and bioactive due to its similarity to the mineral constituents of human bone and teeth [57, 58]. Nanosized HA powder has a high specific surface area which can improve the sinterability and densification of scaffolds. Although pure CS and HA are known as biocompatible materials, the poor mechanical properties of fabricated scaffolds have limited their application [53, 58].

The indirect method uses polymers as a binder with ceramic powders as the main matrix and involves the melting of sacrificial organic polymer to obtain a green part. The green parts are subsequently sintered to produce the final porous ceramic parts [59]. Even though the materials used in this method include both polymer and ceramic as starting materials, the final part is pure ceramic and not a composite.

It has been confirmed that semi-crystalline polymers are preferred over amorphous polymers for use as the binder phase due to their higher density compared with amorphous polymers [59]. However, semi-crystalline shrinks by 4–5 vol% upon solidification, causing component distortion. To reduce distortion, all material is preheated to just below *T*_m_ with SLS heating it in just a small window, i.e., the temperature window between the onset of polymer melting during heating and crystallization during cooling [59, 60]. After SLS the part must be cooled to room temperature slowly.

Fabrication of scaffolds from bioactive glass materials using the indirect method has been reported. Bioactive glass materials with different compositions, e.g., 45S5, 58 S, and 13–93, can be used as scaffold materials [17, 61, 62]. Bioactive glass materials have numerous advantages over other bioactive ceramics like sintered hydroxyapatite. For example, it has been shown that dissolution products from bioactive glasses upregulate the expression of genes that control osteogenesis [63], which explains the high rate of bone formation [64, 65].

#### 2.1.3. Metals

Because metals possess excellent compressive strengths and also high fatigue resistance, porous metallic scaffolds such as titanium (Ti) and tantalum (Ta) and biocompatible alloys such as CoCr and nitinol have been proposed as bone replacement materials, but unlike bioactive ceramics or biocompatible polymeric scaffolds, biomolecules cannot be integrated into metallic scaffolds. The lack of degradability of metallic implants restricts the use of these kinds of scaffolds. The main concern with embedding metallic scaffolds is metal ion release into body fluid, leading to sarcoma. Coating the surface of metallic scaffolds with bioactive ceramics such as HA or CS or using surface finishing methods is highly recommended to improve the biological properties of metallic scaffolds [66].

One category of SLS is selective laser melting (SLM), in which very high laser energy is applied to fully melt metals into a solid homogeneous mass. Different CoCrMo alloys meeting the requirements for tissue applications have been investigated to observe the effect of the laser melting process on corrosion and metal release in biologically relevant fluids [67]. The strong temperature gradient as well as the rapid cooling during the laser melting process induces the formation of a fine cellular microstructure with molybdenum (Mo) enriched at the grain boundaries and suppresses the formation of large micron-sized carbides, resulting in higher corrosion resistance compared with cast alloy.

Dental implants have been fabricated from stainless steel and Ti6Al4V and CrCo alloys by the SLM method [11]. For the consolidation of the powders, two different binding mechanisms are used, depending on the materials and alloys. The first mechanism is liquid phase sintering, where a polymer is liquefied by a laser beam with an energy density of 1 J mm^−3^ and acts as a binder for the stainless steel particles. This technique needs an additional heating cycle in which the polymer is burned out and the green part is further sintered and infiltrated with, e.g., bronze to reach a high density. The second technique is used for Ti6Al4V or CoCr alloy and consists of melting the metal powder completely by a laser beam with an energy density of 200 J mm^−3^, avoiding the need for post-processing. Further surface modification of the laser-melted Ti6Al4V alloy has shown improvement in biocompatibility and a reduction in post-implant complications [68]. The alloys fabricated by SLM are functionalized with a pharmaceutically relevant biomolecule (paracetamol) using phosphonic acid–based self-assembled monolayers (SAMs) to be used as a biocompatible coating layer for drug and protein delivery [68].

#### 2.1.4. Composites

Polymers are elastic and have low stiffness, whereas ceramics are rigid and brittle [69]. By mixing ceramics and polymers into composites, the mechanical properties are significantly improved because the problem of brittleness and the difficulty of shaping hard ceramics can be overcome [53, 58, 70].

Numerous studies have been done to evaluate the potential of SLS in producing composite scaffolds containing polymer and ceramic [19, 71, 72]. The main issue for ceramic/polymer composites is the agglomeration of ceramic powders into the polymer matrix. Using SLS for sintering, a mixture of ceramic and polymer powders can solve this problem due to the uniform distribution of ceramic into the matrix. Studies regarding SLS have included sintering hydroxyapatite powders coated with polymeric binders [72, 73].

A study of scaffolds consisting of microspherical calcium phosphate (CP)/poly(hydroxybutyrate–co-hydroxyvalerate)(PHBV) and carbonated hydroxyapatite (CHA)/poly(L-lactic acid) (PLLA) has shown an improvement in biological properties. Laser-sintered HA/polyetheretherketone (PEEK) can also satisfy the requirements of tissues and scaffolds [74]. PEEK, a synthetic polymer, is a semi-crystalline thermoplastic with excellent mechanical and chemical resistance properties, even at high temperatures. Its Young’s modulus is 3.6 GPa, and its tensile strength is 90 to 100 MPa [75, 76]. PEEK has a glass transition at approximately 143 °C and melts at approximately 343 °C. Since PEEK has a much lower melting point than HA, it is possible to induce sintering of PEEK at temperatures near *T*_g_ and to bind and partially expose the HA particles within the sintered PEEK matrix.

Up to now, there have been few works regarding the fabrication of porous CS scaffolds using SLS and enhancing their mechanical properties by adding HA whiskers at the same time. In a previous study, porous scaffolds from CP materials with different weight ratios of TCP/HAP (0/100, 10/90, 30/70, 50/50, 70/30, and 100/0) were fabricated via SLS [77].

### 2.2. Mechanical properties of SLS parts

Depending on the material and physical properties of the final products, various mechanical properties can be obtained for fabricated scaffolds. A high compressive strength of 18.2 ± 1.2 MPa has been reported for laser sintered CS scaffolds with an interconnected pore structure [56]. Shuai *et al* [78] have reported a Vickers hardness of 4.00 ± 0.13 GPa and a fracture toughness of 1.28 ± 0.03 MPa m^1/2^ for a scaffold made from high surface area HA nano powder by using SLS with a laser energy density of 4 J mm^−2^. Shuai *et al* [79] have fabricated scaffolds via SLS of a composite of CS and poly(vinyl alcohol) (PVA). It was reported that the scaffolds could not be fabricated successfully due to decreased fusion between PVA particles when CS was higher than 20 wt%. For scaffolds containing 15 wt% CS, the compressive strength and compressive modulus reached optimum values of 184 ± 15 kPa and 1.6 ± 0.3 MPa, respectively. Tailoring the porous structure and interconnected pore network in the scaffolds has been reported to increase strength. Feng *et al* [80] have been able to successfully fabricate a highly porous structure with a pore size of 0.5–0.8 mm and fully interconnected pore network scaffolds from HA whiskers incorporated into a CS matrix by SLS. They showed that applying SLS could enhance the compressive strength, compressive Young’s modulus, and fracture toughness of CS with HA whiskers ranging from 0 to 20 wt%. Moreover, for scaffolds made with cellulose material, the specimens with lower particle size showed a higher degree of sintering, a significant level of closed pores, and greater mechanical strength [18].

An interesting work done by Gao *et al* [62] has presented the mechanical properties of SLS scaffolds made with nano-58 S bioactive glass/graphene composite. Recently graphene, a 2D single layer of sp^2^ carbon atoms, has attracted great interest for producing the next generation of nanocomposites used in scaffold fabrication [81]. Due to its superior biocompatibility and mechanical properties, graphene can be used in small amounts as a reinforcing phase in composites. The optimum compressive strength and fracture toughness of the 58 S/graphene scaffolds reached 49 ± 3 MPa and 1.9 ± 0.1 MPa m^1/2^ with a graphene content of 0.5 wt%, indicating significant improvement of 105% and 38% respectively compared with pure 58 S.

Velez *et al* [82] have reported using of 13–93 bioactive glass with a chemical composition of 53% SiO_2_, 4% P_2_O_5_, 20% CaO, 5% MgO, 6% Na_2_O, and 12% K_2_O (wt%) for SLS scaffold fabrication. The compressive strength of the fabricated scaffolds was studied for up to two months when immersed in Dulbecco’s modified eagles medium (DMEM). The compressive strength of the parts decreased from 40 ± 10 MPa in the dry condition and 26 ± 6 MPa after 60 days. Porous biocompatible pure Ti and nitinol (NiTi) alloy was also successfully sintered into 3D scaffold form using a Nd:YAG laser with energy input of 100–300 J cm^−2^ [83]. Nd:YAG lasers outperform CO_2_ lasers with respect to metallic powders due to better absorbance at shorter wavelengths [84]. Applying the same laser energy during SLS resulted in a much smaller sintered depth of monolayers of NiTi powders compared with pure Ti, which causes lower mechanical strength. On the other hand, it is clear that the SLS parameters significantly affect fabricated tissues. As in previous studies, higher scanning velocity as well as laser power resulted in higher mechanical strength, as shown in table 2. Increasing the value of the scanning velocity prevents delaminating between the layers, resulting in enhancement of mechanical strength. This parameter is significant especially for the fabrication of metallic and alloy tissues by the SLM method.

**Table 2.** Mechanical properties and setup parameters of laser-melted Ti6Al4V alloy.

Yielding strength (MPa)	Ultimate strength (MPa)	Scanning velocity (mm s^−1^)	Laser power (W)	Reference
990 ± 5	1095 ± 10	225	195	[85]
1110 ± 9	1267 ± 5	1600	225	[86]

### 2.3. Biological properties of SLS parts: *in vitro* and *in vivo* studies

*In vitro* and *in vivo* tests play an important role in the biological assessment of biomaterials for the fabrication of tissues and scaffolds [87]. A number of architectural characteristics, including porosity, pore size, and permeability, are significant parameters in biological delivery and tissue regeneration. In addition, the materials which are used for tissue engineering must possess a bioactive surface. The ability to control scaffold architecture can provide significant insights into how scaffold architecture and material affect tissue regeneration.

One issue regarding scaffolds made from polymers is the hydrophobic nature of their surface, which results in the negligible availability of bioactive sites [58]. The presence of bioactive binding sites is necessary to induce cell–scaffold adhesion. Chen *et al* [88] have reported a surface modification of PCL scaffolds made by SLS via immersion coating with collagen and gelatin. The collagen-modified scaffold was the best for cartilage tissue engineering in terms of cell proliferation and extracellular matrix production [89]. PCL scaffolds fabricated by SLS can serve as osteoblast or osteogenic scaffolds. They are appropriate scaffolds for the proliferation of adipose-derived stem cells (ASCs) [90]. The addition of bioactive ceramics to hydrophobic but biocompatible polymers is considered beneficial since it reduces the hydrophobicity of the polymer; therefore, it is more favorable for cell attachment and accelerates degradation. The effect of HA addition to a matrix of PCL on MC3T3 osteoblast activity has been examined [71]. The proliferation of adhered cells and the formation of a cell layer on selective laser sintered composites of PCL/HA were observed, and osteoblasts were also encapsulated within the micropores of the struts. The cross sectional images from *μ*CT confirm a remodeling of up to ∼400 *μ*m into the microstructure of the struts. Alamar blue and alkaline phosphate activity (ALP) assays revealed that in general, in the initial period composites with lower HA content (15 wt%) showed better metabolic activity compared with those having higher HA content; however, by day 14 the performance of the two compositions was equal [71].

Das *et al* have fabricated scaffolds from Nylon-6 by SLS [91]. Biocompatibility tests showed that Nylon-6 scaffolds fabricated by SLS support cell viability very well. To investigate the biocompatibility of scaffolds, cells were either in direct contact with the Nylon-6 disks (CoCulture group) or subjected to conditioned media while in contact with the tissue culture polystyrene surface (Conditioned Media group). Two time points were investigated during this study: 3.5 days and 6.5 days in each group. Post-fabrication methods for fabricated scaffolds, e.g., cleaning and treatment, are significant in improving biocompatibility.

*In vitro* tests of 3D scaffolds have demonstrated that the incorporation of CP nanoparticles significantly improves cell proliferation [19] and alkaline phosphatase activity for CP/PHBV scaffolds, whereas CHA/PLLA nanocomposite scaffolds exhibit a level of cell response comparable to PLLA polymer scaffolds. *In vitro* results have also revealed that the addition of bioactive CS ceramic into a PVA matrix (<20 wt%) enhances the bioactivity of scaffolds, i.e., the number of MG-63 cells attached to the surface of the composites increases in the presence of higher amounts of CS in the scaffolds [79].

Williams *et al* [25] have shown that when taking into account external shape and internal architecture, laser sintered scaffolds can support bone regeneration *in vivo* via gene therapy. Histological evaluation and *μ*CT data show that the interior pore architecture of laser sintered PCL scaffolds can induce bone generation *in vivo*. Lohfeld *et al* [92] have proposed the use of a biocomposite blend comprising PCL and TCP prepared by SLS. *In vivo*, a PCL/TCP composite scaffold showed inferior behavior compared with the reference material (*β*-TCP) with respect to a critical size defect regarding the promotion of bone regeneration, scaffold degradation, and inflammatory reaction. Saito *et al* [93] have examined the effect of biomineral coating on bone regeneration for laser sintered PLLA and PCL scaffolds with the same porous architecture. As a result of bone ingrowth analysis after subcutaneous implantation into mice, coated scaffolds encouraged more penetration of bone interior to the scaffolds than uncoated scaffolds. Cross-sections of the biomineral-coated scaffolds showed good bone contact with the biomineral coatings as well as more bonelike tissue formation, indicating that the biomineral coatings supported direct bone formation rather than fibrous tissue formation.

More studies regarding the biological properties of laser sintered tissues and scaffolds from different materials are summarized in table 3.

**Table 3.** Summary of mechanical and biological properties of laser sintered tissues and scaffolds.

Material	Setup parameters	Physical properties	Mechanical properties	Biological properties	Illustration of final part	Cell images	Reference
PCL^a^	Laser power: 3 W Scanning speed: 3800 mm s^−1^	Porosity: 85% Micropores: 40–100 micrometers	Tensile strength: 0.43 ± 0.15 MPa Compressive strength: :345 kPa	A high density of cells was observed on the scaffold after 6 days.	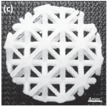	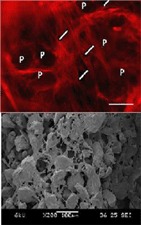	[46]
PCL	Laser power: 1 W Scanning speed: 500 mm s^−1^	Porosity: 83% Micropores: 300–400 micrometers	—	The porcine adipose-derived stem cells (pASC) proliferated well and differentiated into osteoblasts successfully in the scaffold.	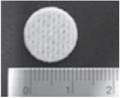	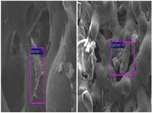	[90]
PCL	Laser power: 3 W Scanning speed: 3810 mm s^−1^	Porosity: 40–84%	Tensile strength: 17–5.03 MPa Compressive strength: 2.74–5.95 MPa depending on porosity and polyhedral model	A confluent monolayer of cells with an elongated morphology could be observed on the wells fed with the scaffold extract.	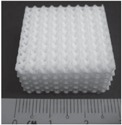	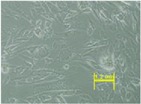	[94]
CP /PHBV CHA/PLLA^b^	Laser power for PHBV: 14 W CP/PHBV: 15 W PLLA: 13 W CHA/LLA: 15 W Scanning Speed: 1257 mm s^−1^	Porosity of the PHBV polymer scaffolds: 64.6 ± 2.0% CP/PHBV scaffolds: 62.6 ± 1.2% PLLA polymer scaffolds: 69.5 ± 1.3% CHA/PLLA scaffolds: 66.8 ± 2.5%	Compressive strength: PHBV: 0.47 MPa CP/PHBV: 0.55 MPa PLLA: 0.51 MPa CHA/PLLA: 0.64 MPa Compressive Young’s modulus: PHBV: 4.9 MPa CP/PHBV: 6.6 MPa PLLA: 5.9 MPa CHA/PLLA: 6.2 MPa	All scaffolds were facilitated proliferation of and ALP expression by SaOS-2 cells. Viability assays of SaOS-2 cells after 3 days of culture on sintered scaffolds	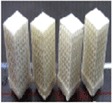	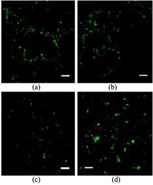	[19]
HA/*β*-TCP^c^	Laser power for PHBV: 14 W CP/PHBV: 15 W PLLA: 13 W CHA/LLA: 15 W Scanning Speed: 1257 mm s^−1^	Porosity: 61% Interconnected macroporous structure of the scaffold with a rectangular pore size range of 0.8–1.2 mm	Fracture toughness: 1.33 MPa m1/2 Compressive strength: 18.35 MPa	MG63 cells exhibited elongated and flattened morphology on the TCP/HAP scaffolds, and the cells were connected with cellular micro-extensions	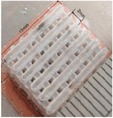	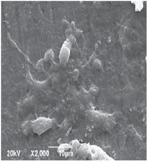	[77]
Forsterite-based scaffolds with 20% nano-58S bioactive glass	Laser power: 9.0 W Scan speed: 100.0 mm min^−1^	Interconnected porous scaffold with pore size 0.5 to 0.8 mm	Compressive strength: 43.91 MPa	Cells attached and spread well on the forsterite /nano-58S	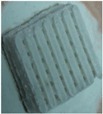	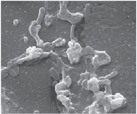	[95]

a Polycaprolactone (PCL).

b Calcium phosphate (CP)/poly(hydroxybutyrate–co-hydroxyvalerate) (PHBV) and carbonated hydroxyapatite (CHA)/poly(l-lactic acid) (PLLA) nanocomposite.

c hydroxyapatite (HA) and *β*-tricalcium phosphate (*β*-TCP).

## 3. Inkjet 3DP technology

**Figure 2.**
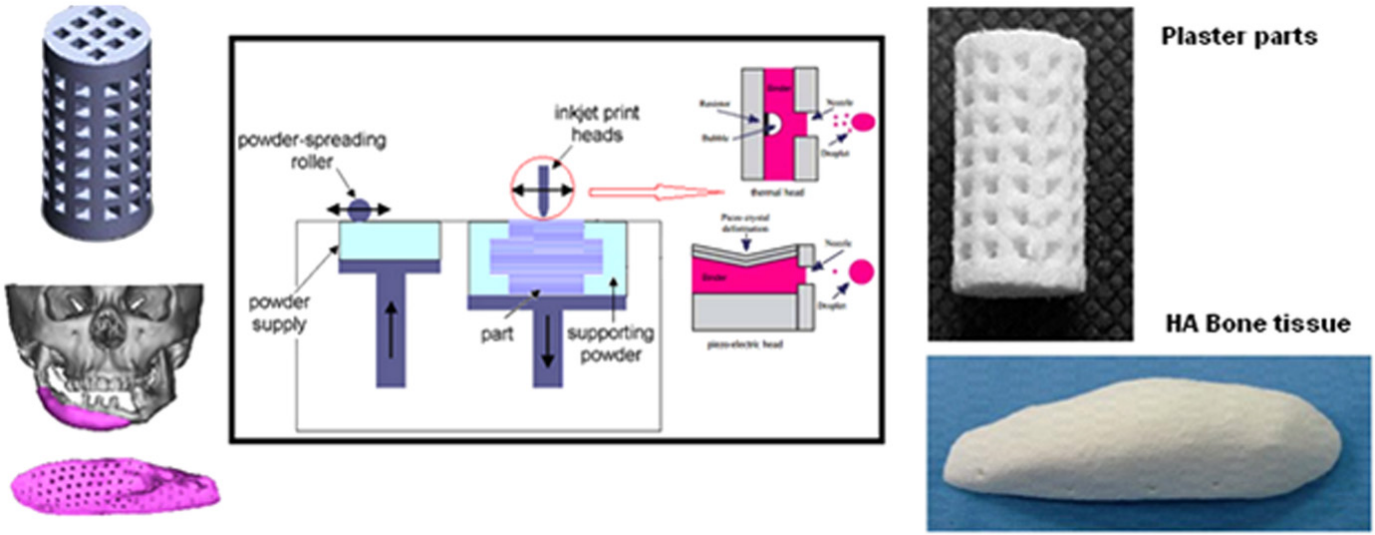
Layout of the inkjet 3DP process. Reproduced with from H Saijo *et al* 2009 *J. Artif. Organs* **12** 200–5, with kind permission from Springer Science and Business Media and A Farzadi *et al* 2014 *PloS One* **9** e108252 under a CC BY 4.0 license.

The binder jetting process is another AM technique which employs inkjet head (IJH) technology for processing materials. In this system, the head prints a liquid binder onto thin layers of powders based on object profiles that have been generated by software [96]. Two kinds of drop-on-demand (DOD) heads can be used in IJH systems: piezoelectric and thermal heads. The main difference between these two heads is their performances. In thermal systems there is a heating element as a thin-film resistor. When an electrical pulse is applied at the head, a high current passes through this resistor and the fluid in contact with it is vaporized, forming a vapor bubble over the resistor. This vapor bubble expands in the fluid reservoir, and the increased pressure causes a droplet to be ejected through the nozzle [97]. In the piezoelectric head system, a volumetric change in the fluid reservoir is induced by the application of a voltage pulse to a piezoelectric material element that is coupled, directly or indirectly, to the fluid. This volumetric change causes pressure/velocity transients to occur within the fluid, and these are directed to produce a drop that issues from the nozzle [98]. Figure 2 shows a layout of the inkjet printing process using both thermal and piezoelectric heads.

Whether to use thermal or piezoelectric inkjet printers depends on the desired properties of the final part. Each inkjet technique has some points which can be categorized as availability, printing speed, accuracy of printed parts, and functional cost. Thermal inkjet printers have some advantages, including availability, higher print speed, and lower cost of parts fabrication compared with piezoelectric inkjet printers [101]. However, the risk of exposing the binder to thermal stress, low droplet directionality, and nonuniform droplet size poses considerable disadvantages with respect to the use of these printers.

On the other hand the advantages of piezoelectric inkjet printers include the capability to generate and control uniform droplet size and ejection directionality as well as to avoid exposure of the binder to heat stressors [102]. The shear stress imposed on the binder at the nozzle tip wall can be avoided by using an open-pool nozzleless ejection system which can also avoid the drawback of nozzle clogging. Adapting piezoelectric printers for less viscous binders in terms of lowering the frequency and power would be challenging since leakage and mist formation during printing may blur the pattern [103, 104].

As the precision of fabricated models strongly depends on the velocity, initial size, and path of the droplets, it is essential to control the parameters, including nozzle diameter, binder properties, and resonance frequency of the head, which have a direct and indirect effect on these terms [102].

### 3.1. Commonly used materials in inkjet 3DP

In general, a wide range of powders including ceramics and polymers can be processed by inkjet 3DP; however, binder selection is a key factor in successful part fabrication. This section provides a detailed discussion of the existing powders and binders which are used for the fabrication of tissues and scaffolds.

#### 3.1.1. Binders

**Figure 3.**
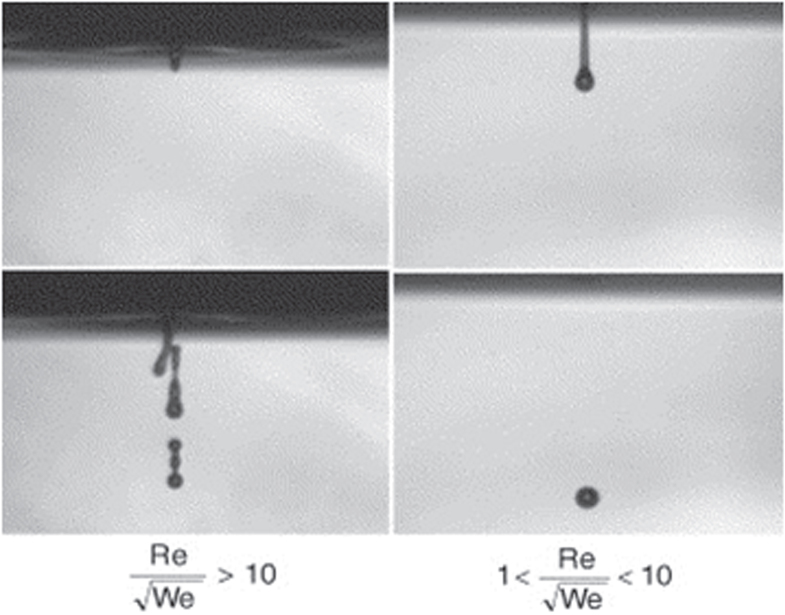
Ejection images of suspensions showing the effect of the ratio of 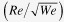. Reprinted from R Noguera *et al* 2005 *J. Eur. Ceram. Soc.* **25** 2055–9, Copyright 2005, with permission from Elsevier.

The materials used as a binder must have suitable properties to prevent spreading from nozzles. To adjust the fluid properties of the organic suspensions to be compatible with the type of printing head, the viscosity and surface tension must be 5–20 Pa.s and 35–40 mJ N^−1^, respectively. To obtain the aforesaid range, the ratio of 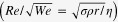 should be between 1 and 10, where *Re* is the Reynolds number 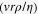 and *We* is the Weber number 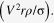 The values 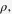 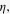 and 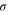 are the ink density, viscosity, and surface tension respectively. *V* and *r* are droplet velocity and radius respectively [105–107]. When this ratio is too small, viscous forces predominate, which implies high pressure for ejection; inversely, if this ratio is too large, a continuous column is ejected that can lead to the formation of satellite drops behind the main drop. Figure 3 shows the different cases observed according to the value of 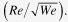 The binder concentration also plays an important role in inkjet 3DP in achieving the desired dimensional precision [108]. Three different types of binders are commonly used in the inkjet 3DP method: water-based binders such as certain commercial ones (e.g., ZB54, Z Corporation) [100], phosphoric acid–based and citric acid–based binders [109], and polymer solution binders such as PVA and poly(D,L-lactic acid) (PDLLA) [110]. Depending on the type of binder, particles are bonded as the result of adhesive forces or a hydraulic setting reaction; i.e., phosphoric acid can react with tricalcium phosphate powder to produce a matrix of dicalcium phosphate dehydrate.

Although polymeric binders have been widely used to fabricate ceramic parts, the final products suffer low resolution and mechanical strength. Using an acid binder solution has been suggested to improve the resolution and mechanical properties of the printed parts [111]. Lyophilized bovine dermal type I collagen has been added to the phosphoric acid binder to improve the bone healing efficacy of the 3D printed scaffolds. The main concern in using collagen in the binder is the increase in viscosity. To cope with this problem a thermal head with a larger valve diameter must be used, which leads to decreased print resolution [112].

#### 3.1.2. Powders

Flowability of powders is an essential parameter for 3DP processing. Sufficient flowability of powders allows the roller to build up thin layers, leading to high 3DP resolution. Too little flowability decreases fabrication resolution due to insufficient recoating. On the other hand, very high flowability does not provide sufficient powder bed stability for 3DP.

Wettability of particles is another factor in 3DP processing. The volume of binder distributed into the powder bed and also the amount of binder absorbed by the powders determines the resolution (voxel size) and mechanical properties of the parts. It has been confirmed that too-low wetting of fine powder particles results in powder bed rearrangement that is possibly detrimental to further 3DP, and too-high wetting and slow powder reaction will reduce the smallest feature size [113–115]. The particle size of powders also has an effect on the mechanical strength of the printed parts. Changing the powder particle size alters the pore size distribution within the powder bed, which influences the drop penetration behavior of a water-based binder [116].

For powder materials, a broad range of polymers, ceramics, and composites can be applied in the field of tissue engineering. As has been previously described the binding mechanism of bioceramic powders used in binder jetting systems is based on the hydraulic setting reaction [117–119]. When dry hydraulic cement is mixed with water, chemical reactions happen in the composite which cause the formation of a firm ceramic-based composite. Because of the nature of the compounds formed in these reactions, they are insoluble in water. This means that the hardened cement will retain its strength and hardness even if immersed in water.

CP has been widely applied in inkjet printing [73, 120]. CP powders can be bound by aqueous (often acidic) binder solutions through a dissolution–precipitation reaction [121]. Solution of a soluble polymeric binder [122, 123] can be used for wet ceramic particles and can glue them together through drying. After the printing process, functioned parts are depowdered and the organic binder removed during sintering [123–125]. Table 4 summarizes the most commonly used powder materials and binders for the production of tissues and scaffolds.

**Table 4.** Powders and binders used for tissue engineering.

Material	Particle size (*μ*m)	Binder	Reference
Plaster-based powder	∼27 (d_50_)	Water-based solution with 2-pyrrolidone	[100]
High-density polyethylene (HDPE)	80–100	Maltodextrin + poly(vinyl alcohol) + lecithin	[126]
Polyethylene + maltodextrin	100–150	Distilled water	[127]
Cornstarch + Dextran + Gelatin	–	Distilled water + blue dye	[128]
TCP + TTCP^a^	10–20	10–20% phosphoric acid	[129]
*β*-TCP	16 (d_50_)	25% oxalic + tartaric acid	[130]
*α*-TCP	30	5% sodium chondroitin sulfate	
		12% disodium succinate	[131]
		83% distilled water	
Calcium silicate	0.3–5	12% polyvinyl alcohol solution	[110]
CP	30–50	8.75% Phosphoric acid	[132]
	50–150		
HA + CaSO_4_	<20 (d_90_) ≥ 20 (d_10_)	Commercial water–based (ZB7)	[133]

a Tetracalcium phosphate (TTCP)

### 3.2. Mechanical properties of inkjet 3DP parts

Improving the mechanical properties of porous parts is a challenge in inkjet 3DP. In some cases, to reach a suitable strength, the scaffolds are sintered after printing. This post-processing exposes the final part to failure due to the burnout of binder which is present or because of high binder concentration. Therefore, the binder concentration must be minimized while still providing sufficient mechanical stability to the printed structure. Moreover, sintering causes a dimensional change in the final part [134]. Tarafder *et al* [135] have reported a significant increase in the mechanical strength of macroporous TCP scaffolds via microwave sintering compared with conventional sintering. Saijo *et al* [99] have fabricated parts with sufficient mechanical strength without using the sintering process. They propose that a reasonable mechanical strength for ceramic scaffolds can be achieved by optimizing the particle size of the powder and the pH and viscosity of the binder.

As mentioned in section 3.1.1, different experiments have been carried out to study the influence of binders on the properties of fabricated parts. In a study of the fabrication of 3D porous strontium-containing mesoporous bioactive glass scaffolds, the 3D printed scaffolds exhibited greater compressive strength (8–9 MPa) than the compressive strength of human trabecular bone (2–12 MPa) [136]. In addition, the mechanical strength of a scaffold could be maintained at approximately 7 MPa after soaking in simulated body fluid (SBF). These results are attributed to the use of aqueous PVA binder, which bonds the ceramic particles together and consequently decreases the brittleness of the scaffolds. Wu *et al* [110] have prepared a *β*-CS scaffold using 12% PVA solution as a binder. The compressive strength and Young’s modulus of printed CS scaffolds with a pore size of 1 × 1 mm and porosity of 65% were 3.6 ± 0.1 MPa and 40 ± 8 MPa. A study of the deformation of scaffolds during the compressive test revealed that the printed CS scaffolds partially maintained a scaffold configuration in the center position and only the border area collapsed. This may be interpreted as the effect of the proper distribution of polymeric binder on the flexibility of the printed scaffolds.

By comparing the compressive strength of CS scaffolds with those using polyurethane (PU) foam and PDLLA solutions to bind the particles, the influential role of binders in inkjet 3DP can be seen. The strengths of scaffolds prepared using PU and PDLLA solutions as binders were 0.3 and 1.45 MPa, respectively, i.e., significantly lower than when using a PVA solution [137].

In the case of acidic binders, Vorndran *et al* [111] have fabricated parts from *β*-TCP as the powder and phosphoric acid as the binder. They improved the compressive strength by adjusting the volume ratio of binder to powder. Compressive strengths of 3.4 and 7.4 MPa were obtained for a binder-to-powder-volume ratio of 2 and 4, respectively. Another study showed that an 8.75 wt% phosphoric acid solution binder can improve mechanical strength while retaining cell viability at 68% ± 6%. As a surfactant, 0.25 wt% Twin 80 was added to the binder solution to improve printability [132].

As mentioned earlier, the size of the powder particles has a direct influence on the mechanical strength of the parts. In HA/CaSO_4_ (calcium sulfate) composites it was confirmed that using very fine HA powders (≤20 *μ*m) leads to a loosely packed powder bed and thus a high level of heterogeneity, which results in slow drop penetration, large drop penetration depth, low wetting ratio, and poor green mass and green strength for the final 3DP components. On the other hand, using coarser HA powders (30–100 *μ*m) can show higher mechanical strength values [133]. Printing the parts along different axes also has an effect on mechanical strength. Composites of HA/PVA as bone tissue have shown different mechanical behaviors along different printing axes [138]. The mechanical strength for *X*-axis scaffolds has been reported as 0.76 ± 0.02 MPa, whereas this value is 0.88 ± 0.02 MPa along the *Y*-axis. Despite exhibiting a higher compressive strength, scaffolds printed along the *Y*-axis have been shown to contain traces of PVA degradation products after heat treatment. Using metal oxide components as a reinforcement agent is also recommended to improve the mechanical properties of bioactive ceramics, especially for hard tissues and implant applications [139, 140]. Moreover, the addition of SiO_2_/ZnO to TCP can increase the mechanical properties of implants. For investigation of this effect, Fielding *et al* [120] fabricated a cylindrical scaffold by binder jetting with the addition of SiO_2_/ZnO. Cylindrical scaffold CAD files were created with interconnected square channels of 1000 *μ*m, 750 *μ*m, and 500 *μ*m sides and 7 mm diameter and 10.5 mm height. The doped fabricated scaffolds, which had less total open pore volume than the pure scaffolds, showed the greatest compressive strength, with the 1000 *μ*m, 750 *μ*m, and 500 *μ*m green channel sizes at 10.21 ± 0.11 MPa, 8.2 ± 0.4 MPa, and 4.34 ± 0.3 MPa, respectively. The pure samples with the green channel sizes 1000 *μ*m, 750 *μ*m, and 500 *μ*m had average compressive strengths of 5.48 ± 0.04 MPa, 2.7 ± 0.2 MPa, and 1.8 ± 0.2 MPa, respectively.

### 3.3. Biological properties of inkjet 3DP parts: *in vitro* and *in vivo* studies

Apart from having good mechanical properties, tissues and scaffolds fabricated by inkjet 3DP must be able to react with cells after implantation. Improving the biological properties (biocompatibility, biodegradability, and cell proliferation) of printed tissues depends on the properties of powders and binders, on pore volume, and also on post-processing of printed tissues.

As previously discussed, in some cases poor mechanical strength of printed tissues can be improved by the sintering process. However, sintering may also compromise biodegradability due to increases in the crystallinity of printed parts, leading to poor resorption by osteoclasts [99]. The binder properties also play a crucial role in the biological properties. The effect of binder solution acidity on the biological properties of printed calcium phosphate scaffolds has been demonstrated by Inzana *et al* [132]. Although higher acidity of binders results in greater mechanical strength of scaffolds, it also increases toxicity. Phosphoric acid of 12.5 wt% almost leads to cell death due to the pH of media falling below 5 [132].

A report by Becker *et al* [141] has presented the prototyping of three scaffolds of HA, TCP, and TCP and bovine HA composites by binder jetting technology. Aqueous solutions of dextrin (20 wt%) and saccharose (2.5 wt%) were used as the binder. After *in vivo* tests and cell seeding, it was concluded that 3D-printed hydroxyapatite and 3D-printed TCP as well as bovine HA blocks are biocompatible for cells derived from a human periosteum.

Studies have shown that zinc oxide has a stimulatory influence on fabricated tissue formation *in vitro* and *in vivo* and also increases the ALP of TCP/zinc oxide composite, which is an enzymatic marker for osteoblastic differentiation [142, 143].

**Figure 4.**
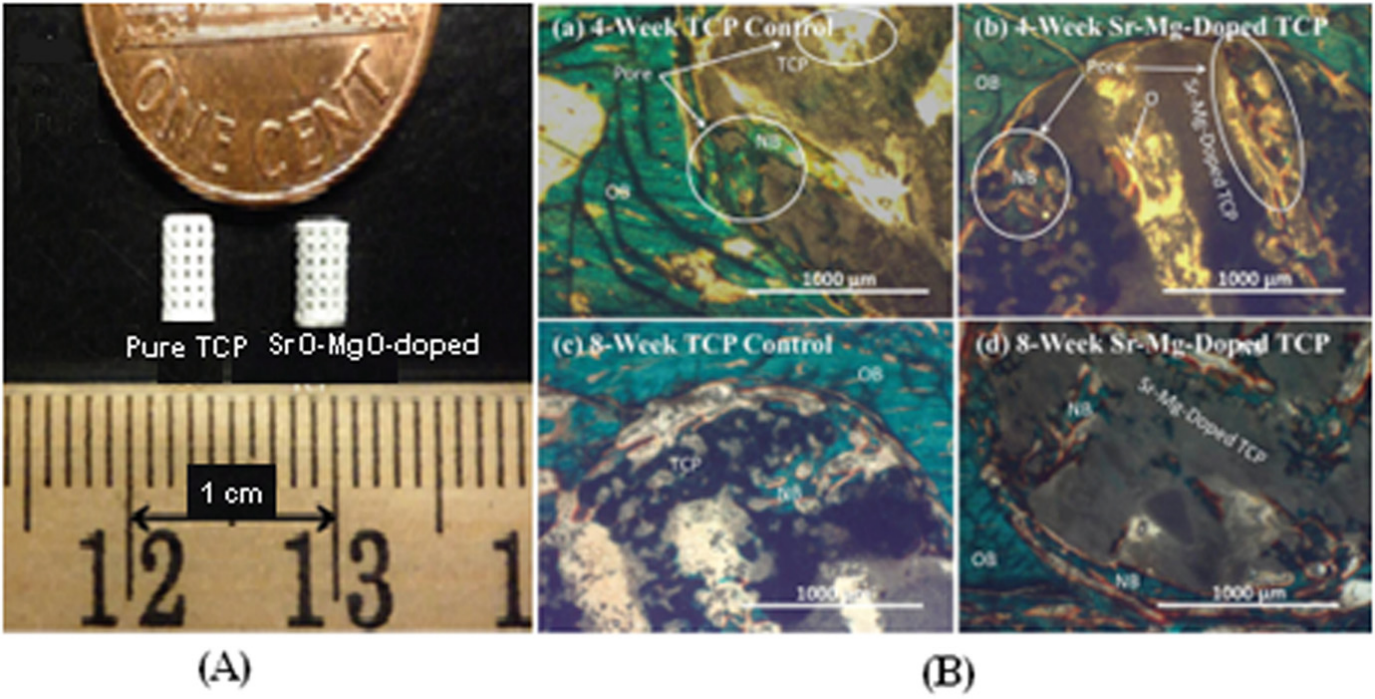
(A) 3D printed tissues; (B) microscopy image of (a) and (c) 3DP pure TCP implants and (b) and (d) Sr/Mg-doped TCP implants, showing the development of new bone formation and bone remodeling inside the interconnected macro and intrinsic micro pores of 3DP scaffolds after four and eight weeks in a rat distal femur model. Modified Masson–Goldner trichrome staining of transverse section. OB: old bone, NB: new bone, O: osteoid, and BM: bone marrow. Color description: dark gray/black = scaffold; orange/red = osteoid; green/bluish = new mineralized bone (NMB)/old bone. Reproduced from S Tarafder *et al* 2013 *Biomater. Sci.* **1** 1250–9, with permission of The Royal Society of Chemistry.

Since bone can grow into pores with a diameter of approximately 300 *μ*m, providing pores of this size or larger is essential for bone grafting. Depending on whether post-processing is used, pores with the desirable geometry can be created by considering the pore size and geometry in the primary design of the structure or can be derived from porogens burned out during sintering. Pore geometry is known to be an important factor in determining bone healing response [144]. The addition of dopants in bioactive ceramics such as TCP can also affect osteogenic differentiation via modification of pore size. Although in many cases cation substitutions such as Na^+^, Mg^2+^, and Sr^2+^ have led to excellent improvement in the biological properties of HA, only a few studies have investigated the effect of cation doping on the 3D interconnected porosity of 3D printed tissues and scaffolds. Both micro and interconnected macropores facilitate the infiltration of osteoprogenitor cells, which emphasizes the presence of multiscale porosity in tissue engineering scaffolds. In a research conducted by Tarafder *et al* [145], the presence of Mg^2+^and Sr^2+^ in a TCP structure and their influence on 3D printed bone tissues led to pore sizes of 245 ± 8 *μ*m and 311 ± 6 *μ*m for doped and pure TCP scaffolds, respectively, which were close to the designed pore size of 350 *μ*m [145]. As shown in figure 4, improvement of bone formation inside macropores (when tested in rat femoral defects) was observed in microwave sintered Mg/Sr-doped TCP tissues. Interconnected pores made by inkjet 3DP result in good cell–tissue reaction, which leads to the development of new bone formation and bone remodeling inside the interconnected macropores and intrinsic micropores of 3D printed scaffolds.

Another *in vivo* study has also revealed the effect of pores on bone formation after implantation, i.e., cylindrical holes 2 mm in diameter running across 3D printed tailor-made bone implants (TIs) showed that bone formation on a larger scale was facilitated [131]. Based on computed tomography (CT) analysis of the skulls of beagle dogs, the volume of the cylindrical holes decreased after the operation, and histological analysis revealed that newly formed bone tissue had invaded the cylindrical holes. Not only can TIs fabricated by an inkjet 3D printer facilitate bone healing due to the excellent natural properties of TCP, but a properly designed hole in the implant structure can also improve bone healing [131].

## 4. Key issues and challenges to clinical applications

AM offers unique advantages with respect to fabrication tissues and scaffolds with a complex external anatomy shape and internal porous structure. Coupling complicated porous 3D design with AM techniques can create a range of bone tissues and scaffolds from various materials. Among the AM techniques, inkjet 3DP and SLS are two powder-based tools which are widely used for biomedical engineering applications.

In the case of SLS, the initial setting, e.g., laser power and scanning speed, is crucial. Without any modification, commercial SLS machines can be used only for small amounts of powdered materials for fabricating specific biomedical applications. Different research groups have begun to optimize the SLS parameters for fabricating special objects with a desired 3D porous architecture in minimum fabrication time and at minimal cost. For tissue engineering, control over mechanical behavior while retaining the designed porous structure is very important. This issue can restrict the use of pure biocompatible polymers. Another disadvantage of the SLS technique for scaffold fabrication is that hydrogels cannot be processed, and it is also impossible to encapsulate cells in scaffolds [84]. The lack of vascularization within scaffolds is still a major concern for scaffolds targeting specific tissue regeneration. Non-fine feature resolution of the SLS technique is a particular drawback which can affect fabricated tissues in terms of cell seeding and growth-factor delivery.

In the case of using inkjet 3DP machines for tissue engineering, although this method can be employed for fabricating tissues with defined shapes and porous architecture from almost all ceramics and polymer materials, the selecting of a suitable binder is still a challenge which needs extensive optimization. Among the binders, acidic ones can provide good mechanical properties for fabricated tissues; however, binder residue in printed structures is difficult to remove and may make tissues toxic. In most cases, post-processing such as sintering is required for printed parts to achieve the desired mechanical behavior. During the sintering process, parts shrink, and unfortunately the shrinkage is not necessarily uniform. The first effect of non-uniform shrinkage on sintered parts is cracking, which make the parts useless. Since the outside part of bone is denser than the inner part, mimicking such structures is very difficult using 3DP, which is the second challenge related to non-uniform shrinkage during sintering [146]. Another post-processing challenge is the removal of loose powders from interconnected pores inside the part. This problem is highlighted for structures with small pores (<600 *μ*m). Trapped powders inside the pores may well sinter with the porous part, making it less interconnected than the designed part. Such problems with loose powders can reduce the dimension of the pores after sintering.

Apart from the issues related to SLS and inkjet 3DP settings as well as selecting suitable biomaterials, clinical usage of AM processed parts is still a big challenge. In fact, there are many obstacles along this long and difficult road.

The gap between the concept and the clinical use of tissue engineering comprises three main factors: the need for understanding native-tissue characterization, the need to incorporate this characterization into tissue design, and finally, the necessity of fabricating tissues based on these design specifications. Despite all the advances in biomaterials science, there are still major gaps in this field relative to the surface chemistry, growth factor release, and mass-transport characteristics that best accelerate a specific tissue formation. Therefore, there are no strategies specifying which material is appropriate for tissues, which linear or nonlinear elastic properties a scaffold should exhibit, which surface chemistry a scaffold should have, or which permeability or diffusion properties a scaffold should demonstrate. In addition to these gaps and challenges, the clinical use of artificial tissues and scaffolds needs volunteer patients for bone tissue replacement surgeries. Because this field is still new and not much clinical surgery has been done, this high-risk surgery might pose challenges after implantation.

**Figure 5.**
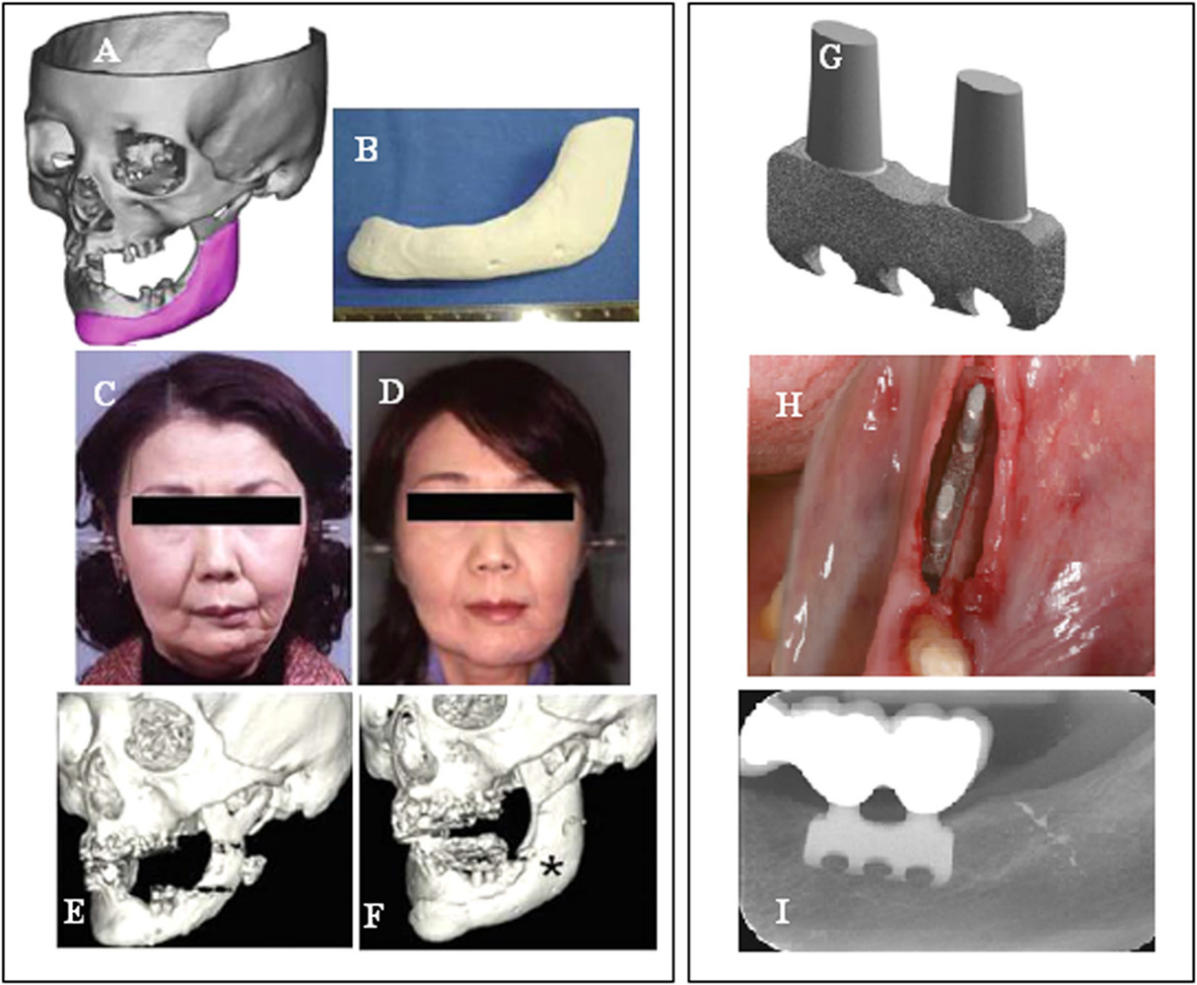
Clinical application of custom-made artificial bone from *α*-TCP using inkjet 3D printing (left) (reproduced from H Saijo *et al* 2009 *J. Artif. Organs* **12** 200–5, with kind permission from Springer Science and Business Media) and custom-made SLS titanium blade implants (right) (reproduced from F Mangano *et al* C 2013 *Lasers Med. Sci.* **28** 1241–7, with kind permission from Springer Science and Business Media). Left: (A) Extraction of the CAD data of the created artificial bone (red) based on a CT image. (B) Macroscopic image of the inkjet-printed custom-made artificial bone (IPCAB). (C) Facial appearance 1 year after surgery. (D) 3D CT image of the left lower jaw before surgery. (E) 3D CT image of the left lower jaw 12 months after surgery. Right: (G) CAD file of the custom-made SLS titanium blade implant. (H) The custom-made SLS blade implant placed in position. (I) The radiographic control two years after implant placement.

Few SLS and ink jet printing products have been used in clinical applications. Most reports have been limited to using models as guide templates for surgery and for *in vitro* and *in vivo* experiments, whereas implantations of scaffolds in the human body are still rare. The union between produced parts and host bones is affected by dimensional compatibility, biodegradability, pore size, and pore interconnectivity. Saijo *et al* [99] have reported a maxillofacial reconstruction by using a custom-made artificial bone made by an inkjet printer. The bone was fabricated with a macropore structure and no sintering process, using *α*-TCP powder with 10 *μ*m particle diameter and a mixture of 5% sodium chondroitin sulfate, 12% disodium succinate, and 83% distilled water as a curing solution. The scaffolds showed rapid union in 10 patients at 12 months after implantation, which can be attributed to the implant macropore structure resulting in rapid cell growth [147]. Recently Mangano *et al* [148] reported a clinical use of SLS titanium (master alloy powder (Ti6Al4V)) blade implants as a non-conventional solution for the prosthetic rehabilitation of extremely atrophied posterior mandibles. Two years after loading, all implants were in good condition and demonstrated perfect aesthetic integration. Compared with conventional approaches such as bone reconstructive surgery, the use of cost-effective SLS implants as a therapeutic treatment can represent an alternative for elderly patients because of lower morbidity. Figure 5 illustrates such custom-made artificial bones fabricated through the use of inkjet 3DP and SLS for clinical applications.

Demand for AM technologies such as SLS and 3DP will increase in the future due to their capability to make custom medical devices that can be tailored for patient-specific and defect-specific clinical needs. Integrating all key points mentioned as well as finding solutions to cope with the challenges and issues are important in guiding the progress of these techniques toward achieving the objective of clinical use.
